# Exercise‐induced dynamic hyperinflation in chronic obstructive pulmonary disease

**DOI:** 10.1113/EP091459

**Published:** 2026-02-05

**Authors:** Rebecca F. D'Cruz, Dominic Wilkins, Caroline J. Jolley

**Affiliations:** ^1^ Lane Fox Respiratory Service, Guys and St Thomas’ NHS Foundation Trust London UK; ^2^ Centre for Human and Applied Physiological Sciences King's College London London UK; ^3^ Department of Respiratory Medicine King's College Hospital NHS Foundation Trust London UK

**Keywords:** COPD, exercise, hyperinflation, physiology measurements, pulmonary mechanics

## Abstract

Chronic obstructive pulmonary disease (COPD) is an inflammatory lung disease caused by inhalation of noxious particles, most commonly cigarette smoking. The consequent changes in airways, lung parenchyma and pulmonary vasculature lead to increased resistive, elastic and threshold loads and impaired capacity of the respiratory muscle pump. COPD is characterized by progressive expiratory flow limitation. During exercise, increases in respiratory rate lead to shortening of expiratory time with consequent gas trapping. The resultant increase in end‐expiratory lung volume is referred to as dynamic hyperinflation. Dynamic hyperinflation leads to further load–capacity imbalance with consequent increased neural respiratory drive to maintain ventilatory homeostasis, which is closely related to exertional breathlessness intensity. Neuromechanical dissociation, resulting in uncoupling of increased neural respiratory drive from ventilatory output, develops due to mechanical limitations on tidal volume expansion and reduced force‐generating capacity of the diaphragm as dynamic hyperinflation progresses during exercise. This review provides an overview of methods of measuring dynamic hyperinflation in COPD and clinical interventions that aim to alleviate lung hyperinflation and improve exercise tolerance.

## Abstract (highlights)

- **What is the topic of this review?**
  This review describes the physiological changes that occur during exercise in chronic obstructive pulmonary disease (COPD), how these can be measured and clinical interventions that alleviate hyperinflation and improve exercise tolerance.
- **What advances does it highlight**
  We provide a review of what is known about dynamic hyperinflation in COPD, advanced physiological measurements to assess it, and clinical advances that aim to reduce hyperinflation and improve the exercise capacity of patients, including bronchodilatation, non‐invasive respiratory support, physical therapy and surgical and endobronchial interventions.

## 1 INTRODUCTION

Chronic obstructive pulmonary disease (COPD) is a heterogeneous lung condition characterized by chronic respiratory symptoms (breathlessness, cough and sputum production) and progressive, largely irreversible airflow obstruction. COPD is common, with an estimated global prevalence of 10.3% (Adeloye et al., 2015). It is a leading cause of morbidity and, excluding coronavirus disease 2019 (COVID‐19), is the third leading cause of death worldwide according to the most recent data from the World Health Organization (2021).

The pathological hallmarks of COPD are small airway inflammation (obstructive bronchiolitis) and destruction of lung parenchyma (emphysema), resulting in poorly reversible expiratory airflow limitation. Lung inflammation in COPD is driven by long‐term exposures to environmental particles and gases, augmented by genetic, developmental and socio‐economic factors (Global Burden of Disease Study Collaborators, 2025). Tobacco smoking, biomass exposure and outdoor air pollution are the most important environmental triggers.

Breathlessness in COPD is typically persistent, progressive, and exacerbated by exercise, and is a major cause of disability and predictor of mortality associated with the disease (Nishimura et al., 2002). Exertional breathlessness limits the ability of an individual to perform day‐to‐day activities, which contributes to the high observed rates of comorbid anxiety and depression (Horner et al., 2023). Reduced exercise capacity also carries important prognostic implications, including a higher risk of exacerbation‐related hospitalization and all‐cause mortality (Spruit et al., 2012). Understanding the pathophysiology of exertional breathlessness in COPD is key to understanding the impact of COPD on health‐related quality of life and the design of effective clinical interventions and treatments. Although there are multiple factors leading to reduced exercise capacity in COPD, including peripheral muscle weakness, heart failure and other extrapulmonary comorbidities, the impairments in respiratory mechanics that result from lung hyperinflation are dominant contributors to exertional breathlessness and are therefore the focus of this review. Here, we provide an overview of the pathophysiology of COPD and exercise‐induced dynamic hyperinflation (DH), methods of conducting physiological assessments to quantify associated load–capacity–drive imbalance and established and emerging therapeutics for relieving DH and improving patient outcomes.

## 2 PATHOPHYSOLOGY OF CHRONIC OBSTRUCTIVE PULMONARY DISEASE

COPD is driven by changes in the airways (predominantly small airways), lung parenchyma and pulmonary vasculature, which arise as a consequence of sustained exposure to noxious particle inhalation. The leading risk factor worldwide is cigarette smoking (Global Burden of Disease Study Collaborators, 2025). Inhalation of cigarette smoke stimulates an inflammatory response, triggering small airway and vascular remodelling, mucus hypersecretion and ciliary dysfunction, and protease‐induced degradation of lung tissue, leading to emphysema (Christenson et al., 2022).

COPD is diagnosed and staged using spirometry testing to demonstrate and quantify the severity of airflow obstruction consequent to increases in airway resistance. The Global Initiative for Chronic Obstructive Lung Disease requires a post‐bronchodilator forced expiratory volume in 1 s as a ratio of forced vital capacity (FEV_1_:FVC) <0.7 to confirm a diagnosis of COPD, together with a compatible clinical history and exposure to relevant risk factors (typically, cigarette smoking). The FEV_1_ is calculated as a value predicted for an individual's age, height and sex to stratify severity (Global Initiative for Chronic Obstructive Lung Disease, 2024).

Increased airways resistance leads to expiratory flow limitation (EFL), the pathophysiological hallmark of COPD (O'Donnell, 2006). EFL is defined as a condition in which, at a given transpulmonary pressure and lung volume, expiratory airflow does not increase despite increased driving pressure (Hyatt, 1983). The driving pressure for expiratory airflow originates in the alveoli (*P*_alv_) and is determined by the intrapleural pressure (*P*_pl_) and the elastic recoil pressure of the lung tissue (*P*_st_). *P*_alv_ is approximately equal to the sum of the pleural pressure and elastic recoil pressure, i.e. *P*_alv_ = *P*_pl_ + *P*_st_. As air flows from the alveoli towards the mouth during expiration, there is a fall in airway pressure owing to airflow resistance along the conducting airways, and at a certain point, the airway pressure falls equal to the surrounding *P*_pl_. This is known as the equal pressure point (EPP). Beyond the EPP, if *P*_pl_ exceeds the pressure within a collapsible airway, the airway will critically narrow and limit expiratory flow (Jordanoglou & Pride, 1968). In health, the EPP typically occurs in the cartilaginous airways. If there are pathological increases in airway resistance, such as in COPD, the pressure drop along the airway is much steeper, hence the EPP will be reached in the smaller, collapsible airways, thereby further limiting maximal expiratory flow (Mead et al., 1967; Voets & van Helvoort, 2013). In emphysema, destruction of alveolar walls leads to a loss of elastic recoil pressure and will further diminish the driving pressure for expiratory airflow, exacerbating EFL. These pathophysiological changes have significant implications for ventilatory mechanics during physical exertion beyond those captured by routine lung function tests. Notably, the severity of breathlessness and quality‐of‐life impairment in COPD are frequently underestimated by simple spirometric indices of airflow obstruction such as percentage predicted FEV_1_ (Jones, 2001).

## 3 RESPIRATORY MECHANICS DURING EXERCISE IN CHRONIC OBSTRUCTIVE PULMONARY DISEASE

The static equilibrium volume of the relaxed respiratory system (*V*_r_) refers to the lung volume at which the elastic recoil pressures of the lungs and the relaxed chest wall are equal in magnitude but opposite in direction. This is the volume at which the forces that expand the lungs are balanced by the forces that contract them, resulting in a stable lung volume, without any active muscle effort. Importantly, functional residual capacity (FRC; the lung volume at the end of tidal expiration) does not always correspond to *V*_r_. For instance, in healthy young individuals during exercise, the activation of expiratory muscles often reduces FRC below *V*_r_ (Henke et al., 1988).

In COPD, progressive EFL and reduced elastic recoil contribute to lung hyperinflation, both at rest and during exercise. An increase in total lung capacity (TLC) beyond the upper limit of normal is referred to as ‘thoracic hyperinflation’. An increase in FRC is termed ‘lung hyperinflation’ (O'Donnell et al., 2015). Increases in resting FRC in COPD have static and dynamic determinants. Static hyperinflation reflects loss of lung elastic recoil, which will increase the lung volume at which lung and chest wall recoil pressures are balanced. DH occurs when expiratory time is insufficient for adequate lung emptying (a consequence of critical EFL) and is exacerbated by increased respiratory rate during exercise, which shortens the time available for expiration within each breath (O'Donnell et al., 2015). Because expiration is terminated prematurely, gas trapping occurs and end‐expiratory lung volume (EELV) rises, resulting in DH.

**FIGURE 1:**
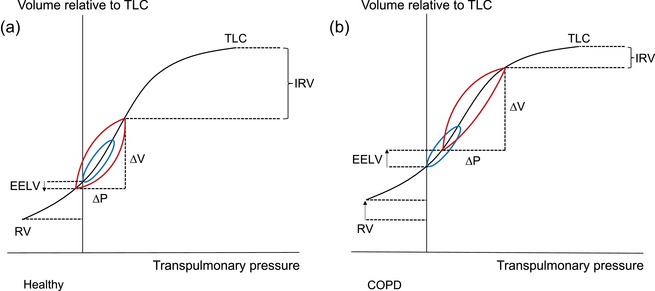
Pressure–volume relationships of the respiratory system in health and COPD. (a) In healthy subjects, breathing occurs on the linear portion of the pressure–volume curve both at rest (blue) and during exercise (red). Dynamic hyperinflation occurs when there is an acute increase in airflow resistance and shortened expiratory time, which leads to an acute rise in end‐expiratory lung volume after each breath. (b) When patients with COPD develop dynamic hyperinflation (during exercise or exacerbations), breathing occurs on the upper, non‐linear portion of the curve, hence greater effort is required to expand tidal volume. Abbreviations: COPD, chronic obstructive pulmonary disease; EELV, end‐expiratory lung volume; IRV, inspiratory reserve volume; Δ*P*, change in pressure; RV, residual volume; TLC, total lung capacity; Δ*V*, change in volume.

DH has several deleterious effects on respiratory mechanics. Inspiratory capacity (IC), the maximal volume of air that can be inhaled after spontaneous expiration to EELV, represents the operating limits on tidal volume expansion during exercise. An increase in EELV leading to a reduction in the IC/TLC ratio, in the setting of EFL, will bring about a reduction in IC and will shift the operating tidal volume closer to TLC at the upper plateau of the pressure–volume relationship of the respiratory system. Progressive increases in EELV during exercise will therefore force COPD patients to breathe on the upper, alinear and less compliant portion of the sigmoidal pressure–volume curve, where elastic load and inspiratory work of breathing are greater (Figure 1). COPD patients with static lung hyperinflation are closer to this threshold at rest.

**FIGURE 2:**
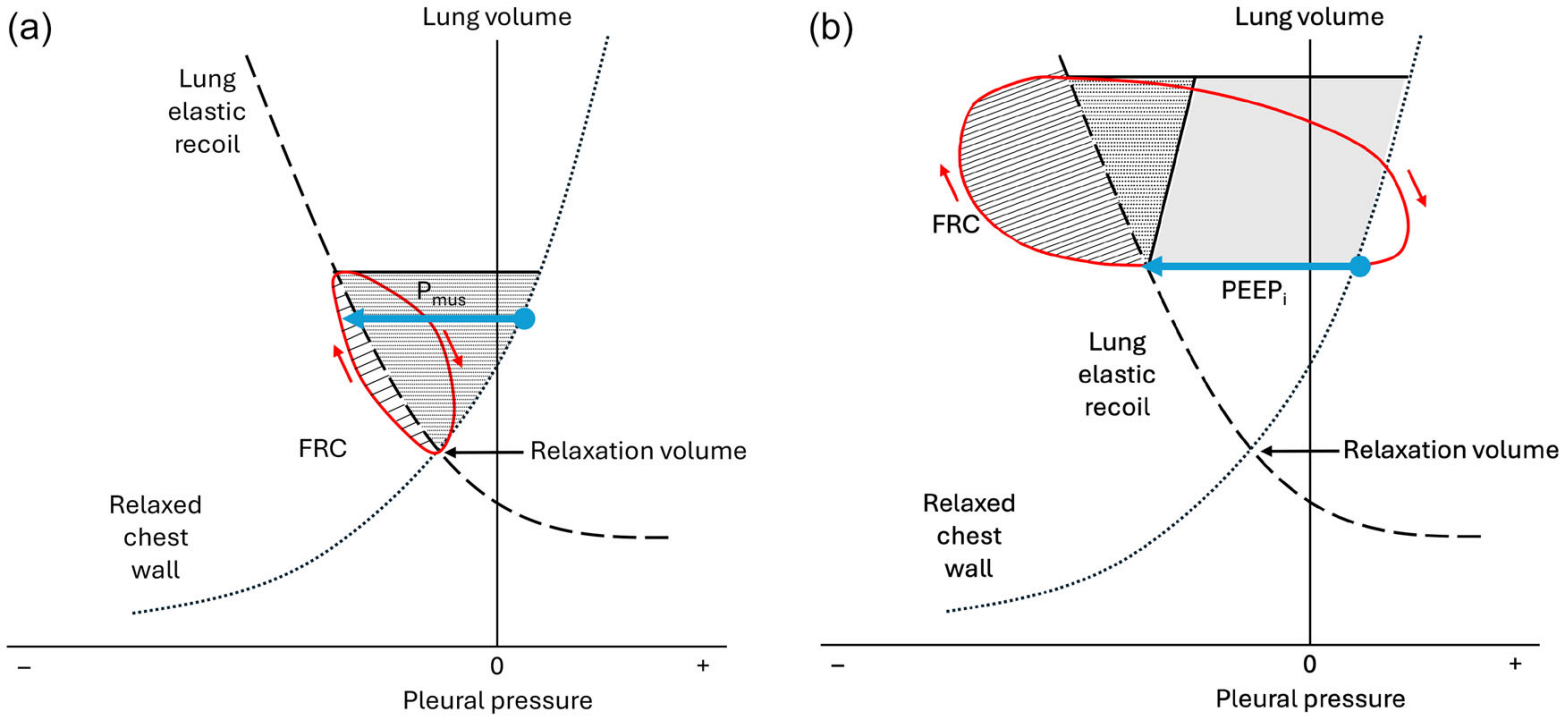
Campbell diagrams plotting lung volume against pleural pressure to illustrate the effects of dynamic hyperinflation. The dashed line is the elastic characteristic of the lung, which has negative recoil pressure, and the dotted line is the elastic characteristic of the relaxed chest wall. The diagonal hatched area is work done against resistance, and the horizontal hatched area is work done against lung and chest wall elastance. (a) A healthy subject, where the relaxation volume of the respiratory system is equal to functional residual capacity (FRC), and *P*_mus_ (blue arrow) is the pressure change generated by inspiratory muscles. (b) Effects of dynamic hyperinflation, where FRC is increased above relaxation volume. Intrinsic positive end‐expiratory pressure (PEEP_i_; blue arrow) behaves as an inspiratory threshold load, which must be overcome by inspiratory muscle work before inspiratory flow can be generated. Adapted from Loring et al. (2009).

Amongst those with severe airflow obstruction, patterns of early and late hyperinflation during exercise have been observed, which can be attributed to the presence or absence of EFL at rest, respectively (Vogiatzis et al., 2005). DH is accompanied by development of dynamic intrinsic positive end‐expiratory pressure (PEEP_i_), as a consequence of EFL and prolonged time constants for lung emptying. PEEP_i_ will further increase the mechanical load during exercise by imposing an inspiratory threshold load, which must be overcome by inspiratory muscles before inspiratory airflow can be initiated (Figure 2; Haluszka et al., 1990). Sliwinski et al. (1998) assessed dynamic elastic work of inspiration (*W*_i_) in patients with severe COPD during incremental cycle exercise until symptom‐limited exhaustion. The total *W*_i_ was divided into two components: *W*_i_,_PEEP__i_, representing the work required to overcome intrinsic PEEP; and *W*_i_,_nonPEEP__i_, representing the work required to overcome the remaining elastic load of the respiratory system. *W*_i_,_PEEP__i_ became an increasingly dominant contributor to total *W*_i_ as exercise intensity rose, accounting for >50% of total *W*_i_ at peak effort. At maximal exercise, *W*_i_,_nonPEEP__i_ was ∼25% higher than predicted based solely on the increase in tidal volume (*V*_T_) (Sliwinski et al., 1998), explained by a marked reduction in dynamic lung compliance during exercise (Suero & Woolf, 1970).

Coupled with functional diaphragm weakness as a consequence of altered diaphragm geometry and sarcomere shortening (Cassart et al., 1997; Polkey et al., 1996), DH will cause an increase in the mechanical load on the respiratory muscle pump and reduce its capacity. This load–capacity imbalance leads to increased neural respiratory drive (NRD) (Moxham & Jolley, 2009). If a critically low inspiratory reserve volume is reached, mechanical constraints on tidal volume expansion limit further increases in ventilatory response (O'Donnell et al., 2001); NRD increases close to maximal, and breathlessness increases to intolerable levels (Faisal et al., 2016; Jolley et al., 2015).

## 4 MEASUREMENT OF DYNAMIC LUNG HYPERINFLATION DURING EXERCISE

Construction of Campbell diagrams to partition and quantify the pressure–volume relationships of the lung, chest wall and the combined respiratory system during exercise (as depicted in Figure 2) represents the gold standard but is a technically challenging method. Surrogate measures are therefore used, including serial measurements of IC and flow–volume loops at rest and during exercise, which can capture the rate and magnitude of exercise‐induced DH (O'Donnell & Laveneziana, 2006). Assuming no change in TLC, measurement of IC at rest and during exercise with subsequent calculation of EELV, end‐inspiratory lung volume, inspiratory reserve volume (IRV) and *V*_T_/IC, allows detection of ventilatory limitation to exercise, and tracking EELV_exercise_ against EELV_rest_ provides an index of the magnitude of DH (Stickland et al., 2022).

In summary, during early exercise, DH allows patients with EFL to increase ventilation with minimal respiratory discomfort and maintain the patency of airways at higher lung volumes, which attenuates EFL and maximizes expiratory flow. However, as exercise intensity and ventilatory demands increase, progressive DH to a critically low IRV will result in constraints on *V*_T_ expansion when *V*_T_ is positioned on the upper, less compliant portion of the sigmoidal pressure–volume curve. Altered chest wall and diaphragm geometry, together with diaphragm sarcomere shortening as a consequence of lung hyperinflation, will place the diaphragm at a mechanical disadvantage and reduce diaphragm force‐generating capacity (Polkey et al., 1996). Together, these changes result in neuromechanical dissociation and neuroventilatory uncoupling, whereby there is an increased work and oxygen cost of breathing with no further increased in *V*_T_ (O'Donnell, Revill, et al., 2001). Exercise‐induced DH imposes constraints on tidal volume expansion in the setting of impaired lung gas exchange, can lead to exercise‐induced oxygen desaturation and carbon dioxide retention (O'Donnell et al., 2002) and can also impact cardiac function (Montes de Oca et al., 1996).

## 5 MEASUREMENT OF NEURAL RESPIRATORY DRIVE AND NEUROMECHANICAL COUPLING DURING EXERCISE IN CHRONIC OBSTRUCTIVE PULMONARY DISEASE

Progressive dynamic hyperinflation during exercise leads to load–capacity imbalance of the respiratory muscle pump, with resultant increases in NRD (Moxham & Jolley, 2009). NRD cannot be measured directly in human subjects, therefore surrogate measures are used to measure this consequence of hyperinflation. Airway pressure during 0.1 s of airway occlusion (*P*_0.1_) can be implemented in spontaneously breathing subjects. The *P*_0.1_ values in stable COPD patients at rest have been cited as −2.5 to −5 cmH_2_O, as compared to −0.5 to −1.0 cmH_2_O in healthy subjects (Tobin, 1998). However, *P*_0.1_ will underestimate NRD in the presence of significant neuromechanical uncoupling, thus is of limited value during exercise amongst individuals with COPD and especially in the presence of DH (Whitelaw & Derenne, 1993).

Respiratory muscle electromyography (EMG) techniques provide indirect, quantifiable estimates of NRD that can be applied to human subjects at rest and during exercise (Hudson et al., 2025; Laveneziana et al., 2019). Crural diaphragm EMG can be measured invasively using oesophageal multi‐pair electrode catheters (oesEMG_di_). The oesEMG_di%max_ (crural diaphragm EMG activity as a proportion of maximal) is higher in COPD compared with healthy individuals, and there is a close relationship between oesEMG_di%max_ and the severity of airflow obstruction and hyperinflation in COPD (Jolley et al., 2009). There is a close relationship between breathlessness intensity and crural EMG_di_ during exercise in COPD, and likewise in health and in restrictive interstitial lung disease (Faisal et al., 2016; Jolley et al., 2015). As previously described, neuromechanical dissociation arises with progressing DH and increasing load–capacity imbalance. Beyond a critical minimal IRV, breathlessness increases rapidly to intolerable levels in association with increased levels of NRD. Qualitative descriptors of breathlessness pertaining to increased inspiratory difficulty (such as ‘unsatisfied inspiration’) have been observed to be associated with neuromechanical dissociation during exercise in COPD (James et al., 2022; Jolley, 2022). Patients with COPD and interstitial lung disease are more likely tto report inspiratory difficulty, with a greater disparity between EMG_di_/EMG_di,max_ and *V*_T_, at end‐exercise compared to healthy controls.

**FIGURE 3:**
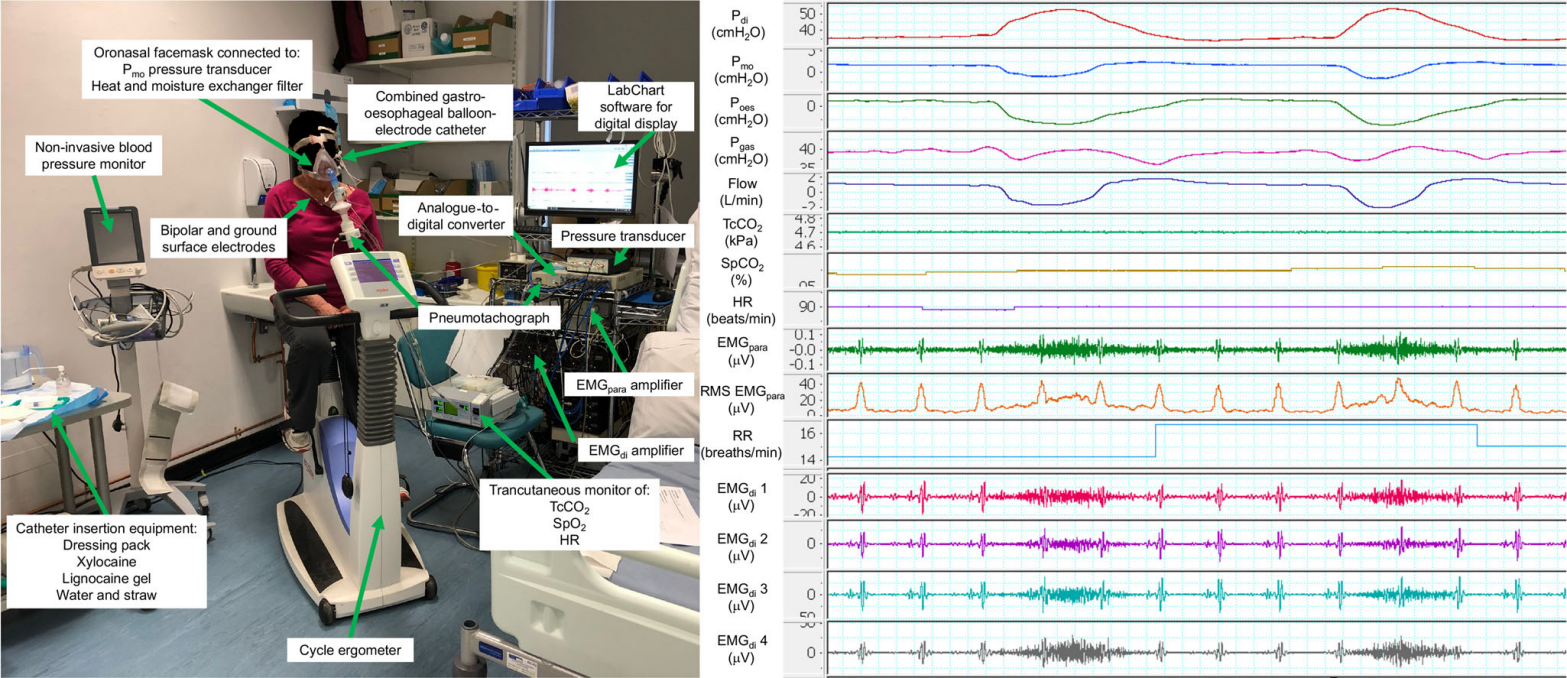
Advanced pulmonary mechanics measurements, including mouth, oesophageal, gastric and diaphragm pressures (*P*_mo_, *P*_oes_, *P*_gas_ and *P*_di_, respectively), airflow, transcutaneous CO_2_ (TcCO_2_), oxygen saturation (SpO_2_), respiratory rate (RR) and parasternal and diaphragm electromyography [EMG_para_, including root mean square (RMS) and EMG_di_] measurements. Analog signals were amplified with a gain of 1000, bandpass filtered between 10 and 2000 Hz and AC‐coupled before acquisition (Bioamp, AD Instruments, Chalgrove, UK), and acquired using a 16‐bit analog‐to‐digital converter (Powerlab, AD Instruments). LabChart software (version 8; AD Instruments) is used to display digital signals. Written patient consent was obtained.

Figure 3 illustrates a laboratory set‐up for measurements of invasive and non‐invasive pulmonary mechanics during cycle exercise, including measurements of airflow, diaphragm pressure [*P*_di_; derived from gastric pressure (*P*_gas_) and oesophageal pressure (*P*_oes_)], transcutaneous gas exchange, heart rate, blood pressure, EMG_para_ and EMG_di_. This arrangement permits measurements of lung volumes and neural respiratory drive to quantify exercise‐induced DH.

**FIGURE 4:**
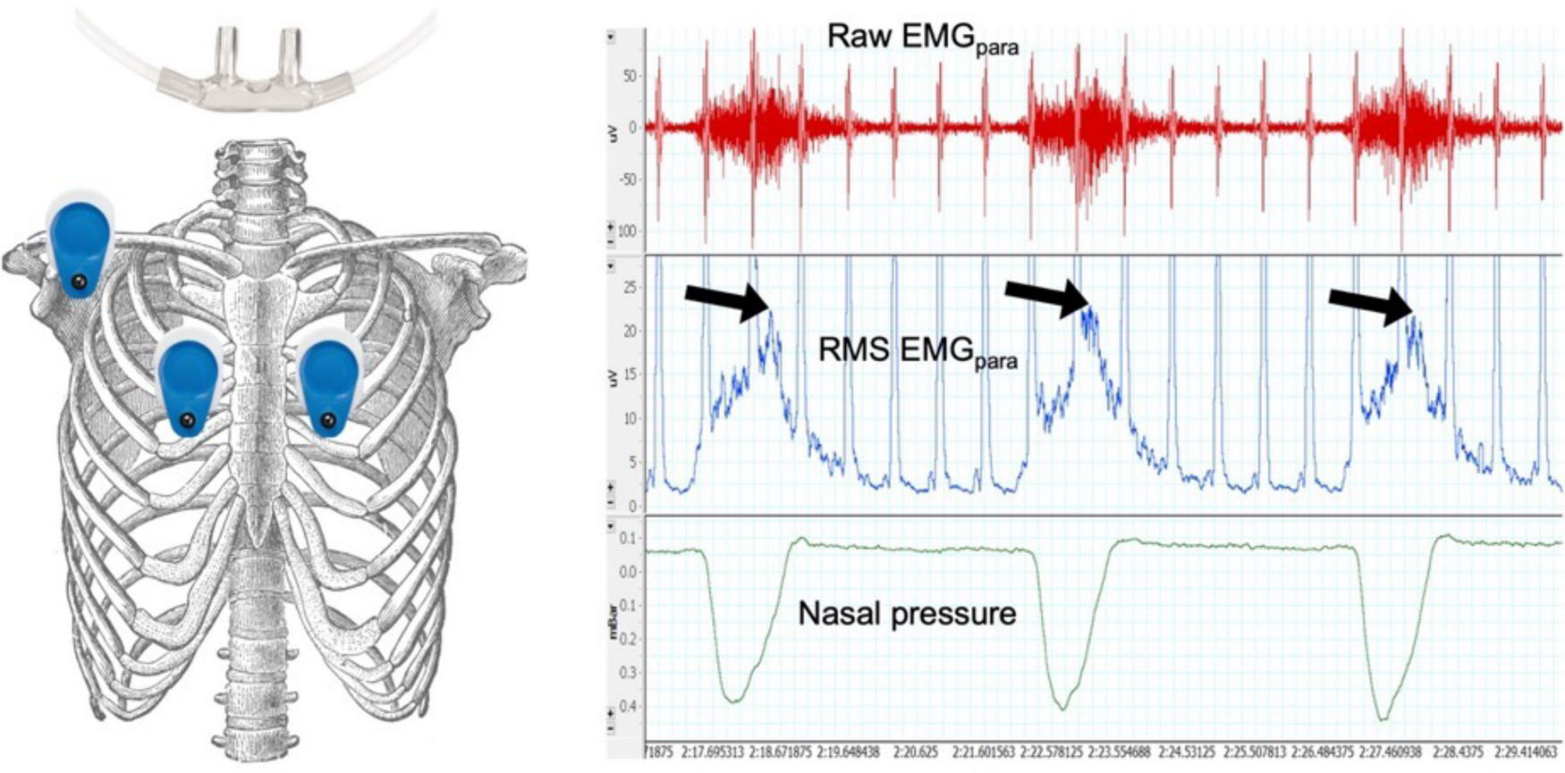
Example trace of a parasternal electromyogram (EMG_para_) measured at rest from a subject with very severe chronic obstructive pulmonary disease. The traces are raw EMG_para_ signal (in microvolts; red), root mean square (RMS) EMG_para_ (in microvolts; blue), nasal pressure (in centimetres of water; green) and respiratory rate (in breaths per minute; pink), with RMS EMG_para_ peaks indicated (black arrows).

Second intercostal space surface EMG enables non‐invasive quantification of parasternal intercostal muscle activity (sEMG_para_; Maarsingh et al., 2002; Figure 4). The advantages of EMG_para_ are its non‐invasive nature and feasibility of application in the hospital and home environments to monitor COPD patients (D'Cruz et al., 2021; Murphy et al., 2011; Suh et al., 2015). Sweat and non‐respiratory muscle artefact can hinder signal quality during exercise.

## 6 INTERVENTIONS

Exertional breathlessness driven by DH and increased NRD has a profound impact on the day‐to‐day physical activity of patients. Breathlessness is frequently disabling, leading to impaired ability to perform tasks independently and altered patterns of behaviour, loss of independence and social isolation. This contributes to higher rates of anxiety and depression observed amongst individuals with COPD compared with age‐ and sex‐matched control subjects (Disler et al., 2014). Pharmacological, surgical and physical therapy techniques may be deployed to ameliorate hyperinflation in COPD to improve exercise capacity.

### 6.1 Pharmacological interventions

Inhaled bronchodilation is a core component of COPD management, delivered typically via inhalers containing a long‐acting anti‐muscarinic and a β_2_‐agonist with or without inhaled corticosteroid (ICS) (Global Initiative for Chronic Obstructive Lung Disease, 2025). Meta‐analyses of randomized clinical trials comparing long‐acting bronchodilatation with β_2_‐agonist or anti‐muscarinic, with or without ICS, versus placebo in patients with moderate‐to‐severe COPD, typically implementing a cross‐over study design, demonstrate prolonged endurance time of 67 s [95% confidence interval (CI) 55–79 s] using cycle ergometry or shuttle walk testing, in addition to reduced breathlessness (Di Marco et al., 2018). A mechanism by which this is achieved is likely to be related to reducing trough IC and modifying DH, since increases in both pre‐exercise IC (157 mL, 95% CI 138–175 mL) and isotime IC (195 mL, 95% CI 162–229 mL) following bronchodilatation were observed (Di Marco et al., 2018).

Inhalation of supplementary oxygen during exercise and exercise recovery has been demonstrated to increase endurance time and reduce breathlessness in COPD (O'Donnell et al., 2001). This is likely to be achieved through reduced respiratory rate and thus increased relative time for expiration to slow DH progression. Oxygen has also been applied during recovery from maximal exercise and is associated with a shorter time to resolution of DH as quantified using IC, compared with air (Stevenson & Calverley, 2004). Oxygen does not appear to influence minute ventilation or breathlessness when delivered during exercise recovery in COPD.

### 6.2 Non‐invasive respiratory support

Delivery of positive airway pressure during exercise in COPD can be applied using non‐invasive ventilation (NIV), whereby inspiratory and expiratory positive pressures are delivered to the upper airway, or using continuous positive airway pressure (CPAP), whereby a fixed pressure is delivered throughout inspiration and expiration. The pressure differential (pressure support) delivered by NIV unloads and assists inspiratory respiratory muscles and reduces work of breathing.

When applied to COPD patients during exercise, NIV has been shown to increase training intensity and exercise capacity and reduce breathlessness compared with sham treatment or no device (Keilty et al., 1994; Kyroussis et al., 2000). It is likely that these improved outcomes relate to alleviation of DH. Indeed, isotime IC has been measured as higher in COPD patients with severe/very severe airflow obstruction undergoing constant‐work‐rate cycle exercise when NIV is delivered in comparison to without, with NIV being associated with longer endurance and lower breathlessness intensity (Dennis et al., 2021). CPAP has been shown to have no effect on exercise endurance and may have a deleterious effect on breathlessness since its adds an expiratory load and may contribute to PEEP_i_, thus increasing work of breathing (Keilty et al., 1994). Despite the physiological and clinical benefits, the main limitations of this technique during exercise are practical constraints imposed by PAP equipment, which typically requires a power supply to a heavy device connected to a circuit and mask.

High‐flow therapy (HFT) is a technique that facilitates delivery of heated humidification, with or without supplemental oxygen, via the nasal passages at flow rates of ≤100 L/min (D'Cruz et al., 2020). HFT has been shown in stable COPD patients and those with acute or chronic respiratory failure to reduce respiratory rate and work of breathing, provide dead space washout and improve gas exchange (Mauri et al., 2017; Moller et al., 2015; Pisani et al., 2017). It may also deliver low positive airway pressure, which increases in proportion to flow rate, but is impacted by whether the mouth is open or closed (Braunlich et al., 2016). HFT has been applied during exercise in subjects with COPD and, although end‐exercise carbon dioxide was lower following exercise with HFT in comparison to room air or conventional low‐flow oxygen, there were no differences in exercise endurance, respiratory rate or breathlessness (Prieur et al., 2019). It may be associated with enhanced resolution of COPD symptoms, particularly airway clearance, during recovery from severe COPD exacerbations (reference D'Cruz et al., 2025).

### 6.3 Physical therapy

Pulmonary rehabilitation is a complex intervention that provides a programme of personalized exercise and disease education aimed at improving symptoms of patients with chronic respiratory disease. High‐quality evidence highlights that pulmonary rehabilitation leads to significant improvements in breathlessness, health‐related quality of life and exercise capacity in stable COPD patients (McCarthy et al., 2015). The mechanism by which these outcomes are achieved is multifactorial and likely to be underpinned by physiological optimization. This has been identified through demonstrable reductions in DH as measured using isotime IC following completion of an exercise programme, in addition to reduced exercise‐induced lactic acid production and improved skeletal muscle oxidative capacity (Maltais et al., 1996; Porszasz et al., 2005). Exercise training may also reduce DH simply because the same external work is undertaken with reduced ventilatory requirement.

Pursed‐lip breathing (PLB) is a technique involving active expiration through partially closed lips which prevents nasal airflow and can generate positive expiratory mouth pressures of 5 cmH_2_O (van der Schans et al., 1995). There appear to be two distinct physiological effects of pursed‐lip breathing. It can lead to: (1) reduced hyperinflation as quantified with EELV, which contributes to increased *V*_T_ and is associated with reduced breathlessness; or (2) increased hyperinflation with no associated improvement in breathlessness (Bianchi et al., 2007). If pursed‐lip breathing is not effective, this is because it is increasing hyperinflation and driving breathlessness.

### 6.4 Surgical interventions

Lung volume reduction can be considered for COPD patients with refractory hyperinflation and breathlessness whose disease management has been otherwise optimized. Surgical approaches to reducing hyperinflation involve resection of non‐functional lung tissue to reduce the severity of hyperinflation and thus improve inspiratory muscle and chest wall mechanics, increase elastic recoil pressure and thus improve expiratory airflow, and improve ventilation and perfusion homogeny to support more effective ventilation. Approaches implemented include open surgery via median sternotomy and stapling or video‐assisted thoracoscopic surgery with laser ablation of non‐functional tissue. Surgical approaches can increase expiratory flow and reduce hyperinflation (measured as residual volume and residual volume as a proportion of total lung capacity) and improve diaphragm capacity (increased sniff *P*_di_, twitch diaphragm pressure,Tw *P*_di_), with associated improvements in health‐related quality of life and exercise capacity (Criner et al., 1998; van Agteren et al., 2016). However, there are associated risks, including postoperative mortality, and morbidity from pneumothorax, air leak, pneumonia or respiratory failure (van Agteren et al., 2016). Consequently, less invasive methods have been developed to induce lung volume reduction, including endobronchial placement of one‐way valves, coils or sclerosant. There have been no studies to date that compare surgical with endobronchial techniques, and long‐term data are limited. The largest evidence base is for endobronchial valves, which are effective at reducing hyperinflation (measured with residual volume) and optimizing diaphragm shape and function, as demonstrated with increased Tw *P*_di_ and increased diaphragm length and zone of apposition on three‐dimensional modelling of computed tomography images. This optimization of respiratory muscle load and capacity is likely to account for the observed improvements in exercise capacity and health‐related quality of life with no apparent difference in early 45‐day mortality compared with control subjects (van Geffen et al., 2019). In eligible cases, the choice of intervention should be determined by a multidisciplinary team, with consideration of individual patient factors and local expertise.

## 7 CONCLUSION

Exercise‐induced dynamic hyperinflation occurs in COPD due to abnormal pulmonary mechanics in the resting state that are compounded by the increasing ventilatory demands driven by physical exertion. DH generates additional resistive, elastic and threshold loads and impairs the force‐generating capacity of inspiratory muscles. This imbalance leads to increased neural respiratory drive in order to maintain ventilatory homeostasis. As elastic loading increases with progressive DH during exercise, the ability to expand tidal volume in response to increased neural respiratory drive becomes limited, which leads to neuroventilatory uncoupling and intolerable breathlessness. A range of invasive and non‐invasive techniques are used to measure load–capacity–drive imbalance and quantify DH. Inspiratory capacity can be measured throughout an exercise protocol and is therefore a valuable surrogate measure of DH, and can be compared with measures of neural respiratory drive and breathlessness to understand exercise capacity in COPD. Clinical interventions aim to optimize pulmonary mechanics to relieve airflow obstruction and reduce lung hyperinflation, thus improving breathlessness and the health‐related quality of life of patients.

## AUTHOR CONTRIBUTIONS

All authors (Rebecca F. D'Cruz, Dominic Wilkins and Caroline J. Jolley) contributed to the final manuscript. All authors approved the final version of the manuscript and agree to be accountable for all aspects of the work in ensuring that questions related to the accuracy or integrity of any part of the work are appropriately investigated and resolved. All persons designated as authors qualify for authorship, and all those who qualify for authorship are listed.

## CONFLICT OF INTEREST

R.F.D., D.W. and C.J.J. have no relevant conflicts of interest to declare.

## FUNDING INFORMATION

No funding recieved.
